# Whole genome sequencing identifies a duplicated region encompassing Xq13.2q13.3 in a large Iranian family with intellectual disability

**DOI:** 10.1002/mgg3.1418

**Published:** 2020-07-26

**Authors:** Sepideh Mehvari, Farzaneh Larti, Hao Hu, Zohreh Fattahi, Maryam Beheshtian, Seyedeh Sedigheh Abedini, Sanaz Arzhangi, Hans‐Hilger Ropers, Vera M. Kalscheuer, Daniel Auld, Kimia Kahrizi, Yasser Riazalhosseini, Hossein Najmabadi

**Affiliations:** ^1^ Genetics Research Center University of Social Welfare and Rehabilitation Sciences Tehran Iran; ^2^ Max Planck Institute for Molecular Genetics Berlin Germany; ^3^ Guangzhou Institute of Pediatrics Guangzhou Women and Children’s Medical Center Guangzhou China; ^4^ Kariminejad – Najmabadi Pathology & Genetics Center Tehran Islamic Republic of Iran; ^5^ Institute of Human Genetics University Medicine Mainz Germany; ^6^ Department of Human Genetics McGill University Montreal Quebec Canada; ^7^ McGill Genome Centre Montreal Quebec Canada

**Keywords:** intellectual disability, whole genome sequencing, Xq duplication, Xq13.2q13.3

## Abstract

**Background:**

The X chromosome has historically been one of the most thoroughly investigated chromosomes regarding intellectual disability (ID), whose etiology is attributed to many factors including copy number variations (CNVs). Duplications of the long arm of the X chromosome have been reported in patients with ID, short stature, facial anomalies, and in many cases hypoplastic genitalia and/or behavioral abnormalities.

**Methods:**

Here, we report on a large Iranian family with X‐linked ID caused by a duplication on the X chromosome identified by whole genome sequencing in combination with linkage analysis.

**Results:**

Seven affected males in different branches of the family presented with ID, short stature, seizures, facial anomalies, behavioral abnormalities (aggressiveness, self‐injury, anxiety, impaired social interactions, and shyness), speech impairment, and micropenis. The duplication of the region Xq13.2q13.3, which is ~1.8 Mb in size, includes seven protein‐coding OMIM genes. Three of these genes, namely *SLC16A2*, *RLIM*, and *NEXMIF*, if impaired, can lead to syndromes presenting with ID. Of note, this duplicated region was located within a linkage interval with a LOD score >3.

**Conclusion:**

Our report indicates that CNVs should be considered in multi‐affected families where no candidate gene defect has been identified in sequencing data analysis.

## Graphical abstract

We report a duplication on the X chromosome encompassing Xq13.2q13.3 in a large Iranian family with X‐linked intellectual disability. There are seven affected males in ones and twos within two generations of the pedigree who presented with a similar phenotype, including ID, short stature, seizures, facial anomalies, behavioral abnormalities, speech impairment, and micropenis.

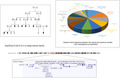

## 1 INTRODUCTION

Intellectual disability (ID) is a commonly encountered problem with the long established role of the X chromosome in its etiology (Ropers & Hamel, 2005). The worldwide prevalence of this disorder is about 1% (Maulik, Mascarenhas, Mathers, Dua, & Saxena, 2011); however, ID is more frequent in societies with high consanguineous marriages. The proportion of X‐linked ID (XLID) has been estimated to be 5%–10% in affected male individuals (Lubs, Stevenson, & Schwartz, 2012). In addition to single gene defects, copy number variations (CNVs) are also a common contributor to XLID, but the extent to which they play a part is currently not fully determined. Based on approximate estimates, 10% of all ID causative CNVs are X‐linked, 70% of which are assigned to duplications and the remainder to deletions (Froyen et al., 2007; Whibley et al., 2010). Regarding this, copy number gains with varying sizes have been reported to cause XLID (Gécz, Shoubridge, & Corbett, 2009; Neri, Schwartz, Lubs, & Stevenson, 2018). A few of those gains serve as pathogenic duplication hotspots, comprising Xp11.22 (Xp11.22 microduplication syndrome; OMIM #300705), Xq21q22 (*PLP1* locus; OMIM *300401, Pelizaeus–Merzbacher disease; OMIM #312080), Xq27 (*SOX3* locus; OMIM *313430, Mental retardation, X‐linked, with isolated growth hormone deficiency; OMIM #300123), and Xq28 (*MECP2* locus; OMIM *300005, Mental retardation, X‐linked syndromic, Lubs type; OMIM #300260) (Gécz et al., 2009). Furthermore, there are other X‐linked regions, reported from a single or few patients, which undergo duplications to a lesser extent (Froyen et al., 2007).

Here, we report a duplication on chromosome X in a family with an established X‐linked pattern of inheritance from a cohort of mostly consanguineous Iranian families.

## 2 MATERIALS AND METHODS

### 2.1 Ethical compliance

A large family with XLID was referred to the Genetics Research Center (GRC), the University of Social Welfare and Rehabilitation Sciences (USWR), Tehran, Iran. After obtaining written informed consent, approved by the Ethics Committee of USWR, the family was enrolled in an ongoing research project aiming to clarify the genetic basis of hereditary ID and patients underwent detailed clinical evaluation.

### 2.2 Next generation sequencing

After chromosome analysis and Fragile X syndrome screening, X‐exome sequencing and whole exome sequencing (WES) were performed for individual (V:1) as described before (Hu et al., 2016, 2019). For whole genome sequencing (WGS) the library was prepared from genomic DNA (gDNA) of individual (V:2) using the Lucigen NxSeq®AmpFREE Low DNA Library Kit, and was sequenced on an Illumina HiSeq X instrument. Quality controlled reads were processed by skewer (v0.2.2) (Jiang, Lei, Ding, & Zhu, 2014) to remove adapters and low‐quality reads were aligned to the human genome build GRCh37 using bwa mem (v7.15) (Li, 2013). Mapped reads were further refined using GATK (v3.8) (Van der Auwera et al., 2013) and Picard program suites (v2.90) ("Picard toolkit," 2019) to improve mapped reads near indels (GATK indel realignment) and improve quality scores (GATK base recalibration), as well as to mark duplicate reads with the same paired start locations (Picard mark duplicates). Germline calls generated using GATK haplotype caller (Van der Auwera et al., 2013) for SNVs and indels were further processed with the addition of functional annotations using snpEff (v4.3) (Cingolani & Platts, 2012) and genomic annotation using Gemini (v0.11.1a) (Paila, Chapman, Kirchner, & Quinlan, 2013). All non‐silent variants were inspected manually for quality control using Integrative Genome Viewers (IGV) (Robinson et al., 2011), and were predicted for functionality using CADD (Rentzsch, Witten, Cooper, Shendure, & Kircher, 2018) and fitness consequence scores (Gulko, Hubisz, Gronau, & Siepel, 2015). Copy number variations on chromosome X were analyzed with CNVkit (v0.9.3) (Talevich, Shain, Botton, & Bastian, 2016) using whole genome argument specification.

### 2.3 Whole genome SNP genotyping and linkage analysis

Genomic DNA samples of 14 individuals (marked by asterisks in the pedigree) were genotyped by Affymetrix Axiom Precision Medicine Research Array (PMRA). Multipoint parametric linkage analysis based on an X‐linked recessive model was done by using the Merlin program (disease allele frequency of 10^−3^, complete penetrance) (Abecasis, Cherny, Cookson, & Cardon, 2002).

### 2.4 Array comparative genomic hybridization (aCGH)

To confirm the results obtained by WGS, whole genome Oligo‐array CGH was performed by using the SurePrint G3 ISCA v2 8x60K platform (Agilent Technologies, Santa Clara, CA, USA). Data were analyzed using the Agilent Cytogenomic software v4.

## 3 RESULTS

### 3.1 Clinical data

**Figure 1:**
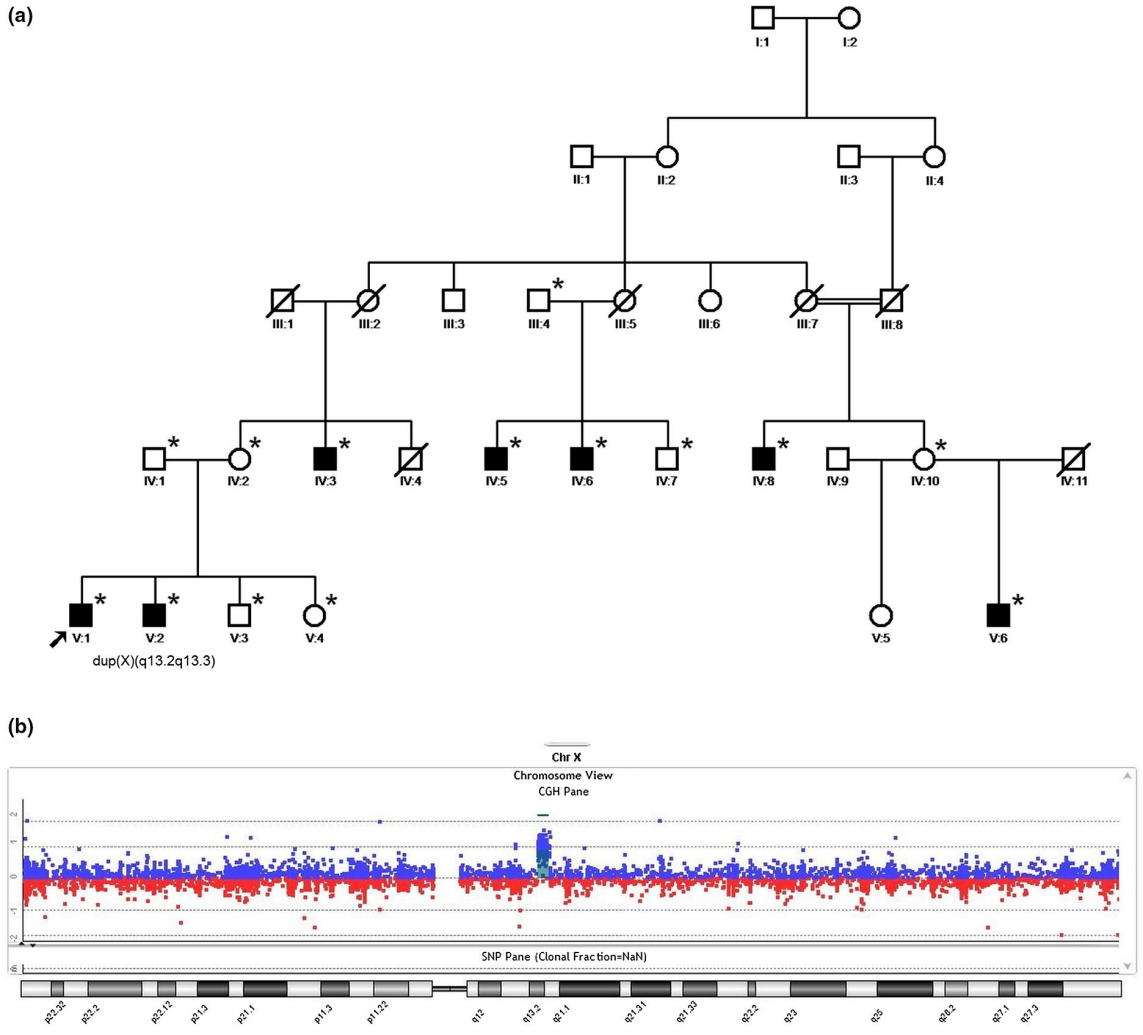
(a) Pedigree of the family (asterisks represent genotyped individuals). (b) The aCGH result for individual (V:2) confirming the Xq13.2q13.3 duplication.

The family originating from the Northern East part of Iran comprises seven affected males in two generations related through the maternal lineage (Figure 1a). The affected males presented with a similar phenotype (see Table S1, column B). The index patient (V:1) and his brother (V:2) were born at term after an unremarkable pregnancy and neonatal period. Both had a normal development with a history of seizures (starting at age 4 years), short temper, anxiety, and self‐injurious behavior. They started to speak first single words at 12 months. Their vision and hearing was normal, and they could walk normally. At 15 and 24 years, height of V:1 was 165 cm (−0.5 SD) and of V:2 168 cm (−1.19 SD); OFC of V:1 was 57 cm (+1.4 SD) and of V:2 59 cm (+2.0 SD). Their facial appearance showed a broad forehead, strabismus, low‐set protruding ears as well as micrognathia, and both presented with micropenis. They had normal gait but developed difficulties with speech articulation and dysarthria after starting to form sentences at 24 months. IQ was not evaluated by standard testing, but cognitive ability appeared to be moderately disabled, as they could understand instructions, count money, follow commands, and indicate their needs by themselves. They showed aggression, hyperactivity, impaired social interactions, and shyness. Bladder and bowel control appeared to be normal. One female sibling (V:4) and the carrier mother (IV:2) had a normal phenotype.

### 3.2 Genetic investigations

Individual (V:1) had a normal karyotype and a negative test for *FMR1* repeat expansion (Fragile X syndrome; OMIM #300624). X‐exome and whole exome sequencing did not provide evidence for a potentially disease‐causing deletion or single nucleotide variant (SNV). Subsequent SNP genotyping followed by linkage analysis delineated a linkage interval on chromosome X (~18 Mb), located between heterozygous SNP markers rs241748 and rs2157410 (GRCh37/hg19), with a significant LOD score of 3.879 (Figure S1). WES data reanalysis did not reveal a candidate gene defect located within the linkage interval, thus, WGS was performed. As a result, a duplication of ~1.8 Mb was detected: seq[GRCh37] dup(X)(q13.2q13.3) chrX:g.72853142_74633399dup, which was located within the linkage interval. Oligo‐array CGH extended this result showing the following imbalance: arr[GRCh37] Xq13.2q13.3(72803969_72804028,72902578_74620919x2,74631526_74631585) in individual (V:2) (Figure 1b) and excluded the duplication in the healthy sister (V:4).

## 4 DISCUSSION

**Figure 2:**
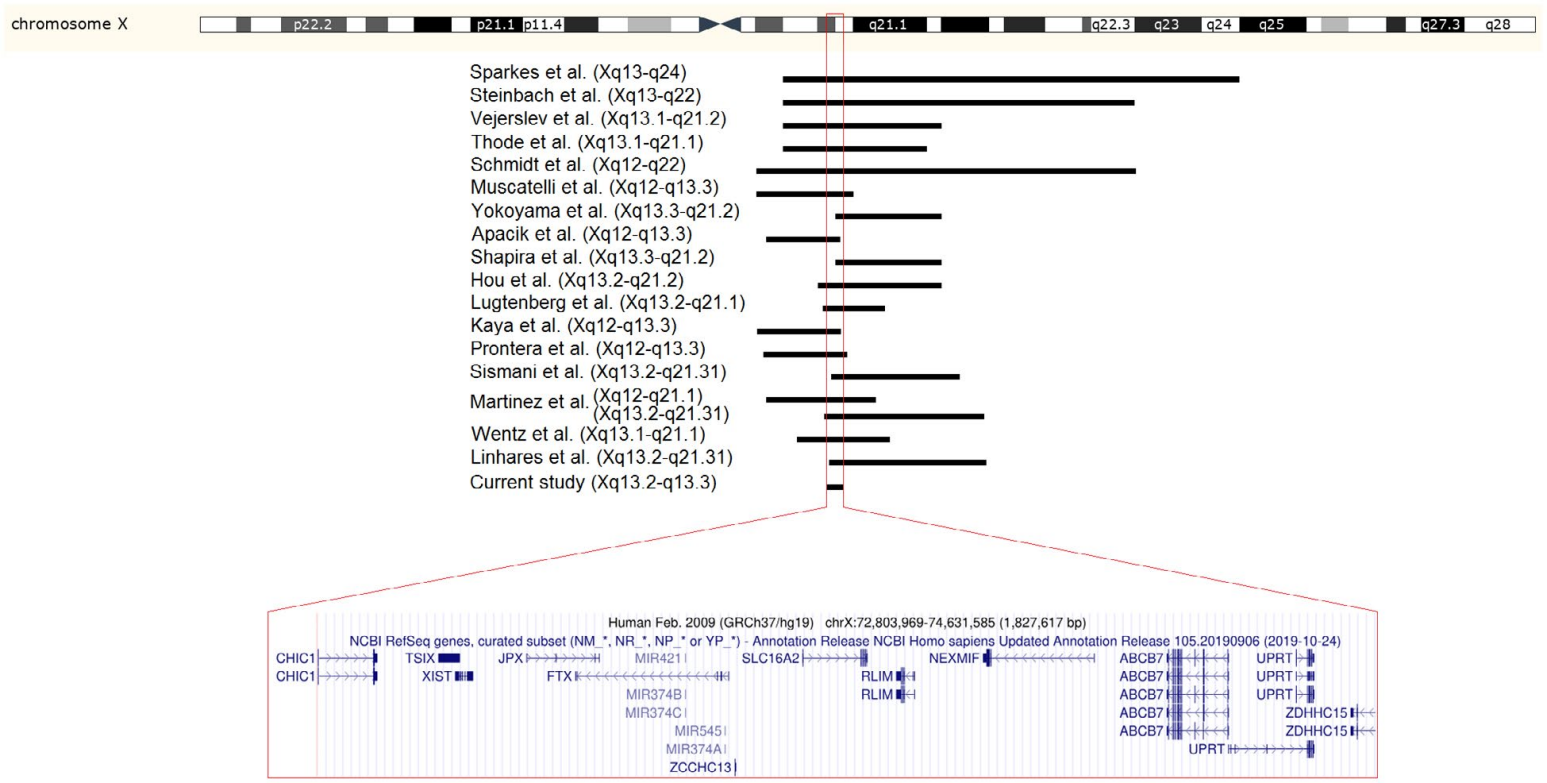
Schematic view of chromosome X with overlapping proximal Xq duplications. Expanded view shows duplicated genes† in the overlapping region (from UCSC Genome Browser http://genome.ucsc.edu/). †NC_000023.10 Reference GRCh37.p13 Primary Assembly.

We report a Fragile X‐negative multi‐affected family with an X‐chromosome duplication in which the patients presented with ID, short stature, seizures, facial anomalies, behavioral abnormalities, speech impairment, and micropenis. Based on WGS and linkage analysis, individual (V:2) carried a duplication at Xq13.2q13.3. The duplicated region contains approximately 40 genes (protein‐coding, pseudogenes, and long noncoding/micro RNAs), comprising 11 OMIM genes, namely *CHIC1* (OMIM *300922), *TSIX* (OMIM *300181), *XIST* (OMIM *314670), *JPX* (OMIM *300832), *FTX* (OMIM *300936), *SLC16A2* (OMIM *300095), *RLIM* (OMIM *300379), *NEXMIF* (*KIAA2022*) (OMIM *300524), *ABCB7* (OMIM *300135), *UPRT* (OMIM *300656), and *ZDHHC15* (OMIM *300576) (Figure 2). Three of the duplicated genes, namely *SLC16A2*, *RLIM*, and *NEXMIF* have ID‐associated OMIM phenotypes: Allan‐Herndon‐Dudley syndrome (AHDS; OMIM #300523), Tonne‐Kalscheuer syndrome (TOKAS; OMIM #300978), and Mental retardation, X‐linked 98 (MRX98; OMIM #300912), respectively. The genes *CHIC1* and *ZDHHC15* are located at the borders of the duplicated region. Loss of expression of the latter, which is involved in neuronal connectivity (Shah, Shimell, & Bamji, 2019), was described in a female patient with severe ID and normal stature; however, another study reported disruption of this gene in a woman with normal cognition, thus, questioning its role in ID (Mansouri et al., 2005; Moysés‐Oliveira et al., 2015).

CNVs, as well as single gene defects, are a common cause of ID. In contrast to autosomes, duplications on the X chromosome have been assigned to play a role equal to or more common than deletions in this disorder (Moey et al., 2016). In this regard, several duplications have been reported in different regions of chromosome X that contribute to XLID, such as Xq21q22 (Mimault et al., 1999), Xq27 (Arya et al., 2019), Xq28 (Sanlaville, Schluth‐Bolard, & Turleau, 2009), Xp11.22 (Froyen et al., 2008), and Xp22.12 (Tejada et al., 2011). There are also duplications encompassing Xp22.3 region (Esplin et al., 2014; Lintas et al., 2015; Pavone, Corsello, Marino, Ruggieri, & Falsaperla, 2018). Concerning the latter, cognitive impairment, behavioral abnormalities, and seizures have been reported in many patients with Xp22.31 duplication; however, the clinical significance of this duplication is controversial (Furrow et al., 2011). Although there is a considerable amount of literature on chromosome X CNVs, duplications, since first reported in the 1980s, are not frequent, particularly those in the proximal long arm (Sanlaville et al., 2009). Some reports have been published on proximal Xq duplications with varying sizes in male patients, the majority of which included the minimum region Xq13.3q21.1 encompassing the ID‐related *ATRX* gene (OMIM *300032) (for details, see Table S1 and references therein). The shared clinical features in 11 patients out of 25 included ID, short stature, and genital abnormalities. Furthermore, autism and/or behavioral disturbances have been reported in 14 patients, 12 of which also showed speech impairment (including those with Xq12q13.3 duplications, see Table S1). The overlapping region in most previous reports encompassed the cytogenetic duplication breakpoints of the Iranian family, i.e., Xq13.2q13.3 (Figure 2). There are some overlapping major clinical features between the affected individuals of this family and TOKAS, which is caused by *RLIM* loss‐of‐function mutations, including ID, short stature, facial anomalies, behavioral abnormalities, and speech impairment (Frints et al., 2019; Tønne et al., 2015). Dosage sensitivity evaluation of the genes in this region has not shown any triplosensitivity for *XIST*, *SLC16A2*, *NEXMIF*, and *ZDHHC15*, although the remaining genes await review (https://www.ncbi.nlm.nih.gov/projects/dbvar/clingen). However, a whole *NEXMIF* duplication, leading to reduced expression, has been reported in a family with XLID and autism (Charzewska et al., 2015). Moreover, duplications comprising some OMIM genes in this region in patients with developmental delay have been reported to ClinVar (e.g., VCV000058643) and DECIPHER (e.g., 345223) databases.

In conclusion, we report on an Xq duplication in a large family with XLID and additional features. More investigations are needed to determine which gene(s) within the duplicated region contributed to the phenotype of the affected individuals and provide a wider perspective on the underlying genetic defect. Our report clearly shows that CNVs should be considered for all families with several affected individuals and no promising single gene variants in WES data re/analysis, even in consanguineous societies. It should also be noted that SNP genotyping followed by linkage analysis is still a powerful tool to narrow down the region of interest in WGS data analysis.

## CONFLICT OF INTEREST

Authors declare no conflict of interest.

## AUTHORS’ CONTRIBUTION

SM: drafting the manuscript, FL: providing linkage analysis, HH: running WES data, ZF: analysis of WES data, MB: interpretation of CNV analysis, SSA: segregation study, SA: providing samples and obtaining the informed consent form, HHR: providing WES data, VMK: providing X‐exome sequencing data, DA: running WGS data, KK: clinical examination and genotype‐phenotype correlation, YR: analysis of WGS data, HN: study design, providing financial support for the project, supervision.

## Supporting information

Fig S1

Table S1

## Data Availability

The data that support the findings of this study are openly available in [ClinVar] at [https://submit.ncbi.nlm.nih.gov/clinvar/], Submission ID [SUB7093314].
