# Supplementary figures and images for: Whole genome sequencing identifies a duplicated region encompassing Xq13.2q13.3 in a large Iranian family with intellectual disability
Source: Mol Genet Genomic Med. 2020 Jul 26;8(10):e1418. doi: 10.1002/mgg3.1418 (PMC7549592; doi:10.1002/mgg3.1418)

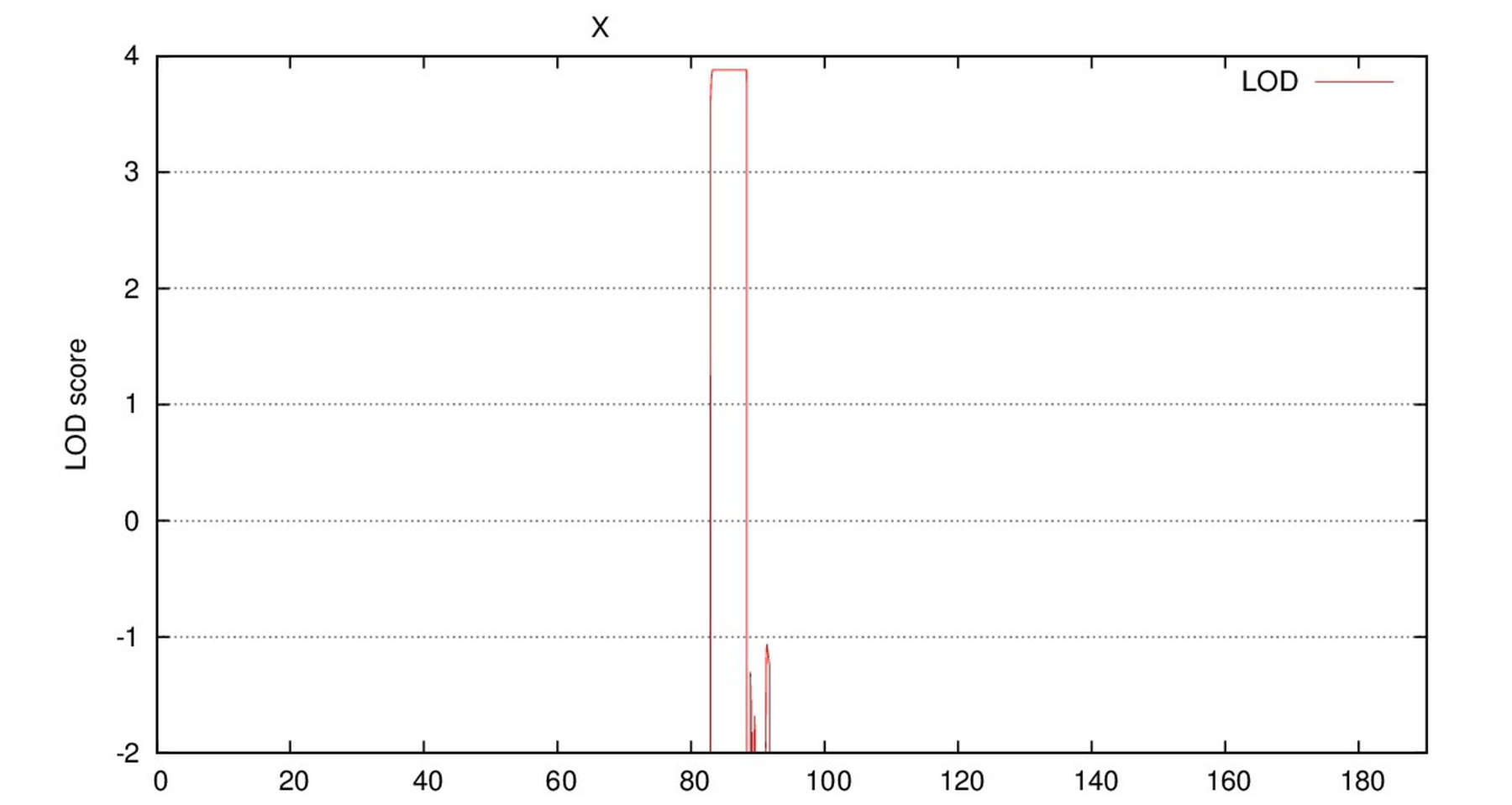

Supplement: Supplementary file 1 — Fig S1 [file MGG3-8-e1418-s001.png]
